# CDX2 expression and perioperative patient serum affects the adhesion properties of cultured colon cancer cells

**DOI:** 10.1186/s12885-020-06941-y

**Published:** 2020-05-14

**Authors:** Johanne Davidsen, Stine Bull Jessen, Sara Kehlet Watt, Sylvester Larsen, Katja Dahlgaard, Tove Kirkegaard, Ismail Gögenur, Jesper T. Troelsen

**Affiliations:** 1 Department of Science and Environment, Enhanced Perioperative Oncology (EPeOnc) Consortium, Roskilde University, Universitetsvej 1, 4000 Roskilde, Denmark; 2 Center for Surgical Science, Enhanced Perioperative Oncology (EPeOnc) Consortium, Department of Surgery, Zealand University Hospital, Lykkebækvej 1, 4600 Køge, Denmark; 3 Department of Clinical Immunology, Naestved Hospital, Ringstedgade 77B, 4700 Naestved, Denmark

**Keywords:** Colon cancer, CDX2, Tumour suppressor, Surgical stress, Metastasis, Cell adhesion, Perioperative phase

## Abstract

**Background:**

Colon cancer is one of the most commonly diagnosed types of cancer with surgical resection of the tumor being the primary choice of treatment. However, the surgical stress response induced during treatment may be related to a higher risk of recurrence. The aim of this study was to examine the effect of surgery on adhesion of cultured colon cancer cells with or without expression of the tumour suppressor CDX2.

**Method:**

We enrolled 30 patients undergoing elective, curatively intended laparoscopic surgery for colon cancer in this study. Blood samples were drawn 1 day prior to surgery and 24 h after surgery. The samples of pre- and postoperative serum was applied to wild type colon cancer LS174T cells and CDX2 inducible LS174T cells and adhesion was measured with Real-Time Cell-Analysis iCELLigence using electrical impedance as a readout to monitor changes in the cellular adhesion.

**Results:**

Adhesion abilities of wild type LS174T cells seeded in postoperative serum was significantly increased compared to cells seeded in preoperative serum. When seeding the CDX2 inducible LS174T cells without CDX2 expression in pre- and postoperative serum, no significant difference in adhesion was found. However, when inducing CDX2 expression in these cells, the adhesion abilities in pre- and postoperative serum resembled those of the LS174T wild type cell line.

**Conclusions:**

We found that the adhesion of colon cancer cells was significantly increased in postoperative versus preoperative serum, and that CDX2 expression affected the adhesive ability of cancer cells. The results of this study may help to elucidate the pro-metastatic mechanisms in the perioperative phase and the role of CDX2 in colon cancer metastasis.

## Background

Colon cancer is the third most commonly diagnosed cancer and the second most leading cause of cancer-related death, accounting for approximately 1 in 10 cancer cases and deaths [1]. Surgical resection of the tumour is the primary choice of treatment but, despite medical and surgical advances, the risk of recurrence in colonic cancer is up to 30% after curative resection [2]. Manipulation of the tumour during surgery results in an increase in the number of circulating tumour cells [3], and the operation can lead to a surgical stress response (SSR) resulting in reduced anti-tumoral defence [4], as well as an increase in factors favouring an oncogenic environment [5]. Excessive stimulation of cytokine production during the SSR is associated with the risk of postoperative metastasis [6], and pro-inflammatory cytokines, such as IL-1 and TNF-α have been shown to stimulate adhesion in circulating cancer cells [2].

Alternations in cell adhesion is believed to be critical in cancer metastasis [5]. For tumour cells to disengage from the primary tumour, adhesion is downregulated through modification of the cadherin-catenin complex [7, 8]. Further, the integrity of tight junctions that maintain cell polarity in normal epithelia is diminished by downregulation of claudins [9]. Attachment of metastatic cancer cells to distant tissues is mediated through expression of selectins [10], integrins [11] and members of the immunoglobulin superfamily [12]. Surgical trauma provokes an inflammatory reaction which results in release of cytokines that are shown to increase adhesion of colon carcinoma cells to metastatic sites [13, 14]. This effect of cytokines on the cell adhesion molecules expressed on cancer cells may contribute to the development of metastasis [15].

The transcription factor Caudal type Homeobox 2 (CDX2) is crucial for the homeostasis of the colonic epithelium [16], and has been shown to be at tumour suppressor [17–20]. Lack of CDX2 expression in colon cancer cells is associated with aggressive clinical behaviour and can be used as an adverse prognostic biomarker [21–25]. CDX2 has been reported to be downregulated in colon cancer cells in the invasive front of the tumour [16, 26]. The downregulation of CDX2, and thereby loss of intestinal identity, has been suggested to be a precursor for metastatic colon cancer to perform epithelial-to-mesenchyme transition (EMT) [16, 26, 27]. As the circulating colon cancer cells establish metastasis they undergo mesenchyme-to-epithelia transition (MET) and CDX2 expression is re-established, allowing for it to be used as a marker to determine the primary tumours colonic origin [26]. Overexpression of CDX2 in colon cancer cell lines has shown decreased mobility and dissemination of cancer cells, further implicating fluctuation of CDX2 expression in the metastatic process [28]. Alterations in CDX2 expression is based on mechanisms such as inflammation and epigenetic regulation, rather than mutations [21, 29].

Through a cell-based assay, measuring cancer cell adhesion in a colon cancer cell culture treated with serum obtained from patients before and after colon cancer surgery, we aimed to examine whether laparoscopic colon cancer surgery affects the adhesion of cancer cells and if CDX2 influences the adhesion abilities of cultured colon cancer cells.

## Methods

### Participants

From January to July 2016, consecutive patients undergoing elective, curatively intended laparoscopic surgery for colon cancer, stage I-III according to Union for International Cancer Control (UICC), at Zealand University Hospital were enrolled in this study. Patients receiving neoadjuvant radio- or chemotherapy, with known immune defects, or previous cancer history, were excluded. All eligible patients received information regarding purpose and methods of the study and were included after giving oral and written consent. The study was conducted in accordance with the Declaration of Helsinki, and the protocol was approved by The Danish National Committee on Health Research Ethics, Region Zealand (file no: 2008-58-0020) and approved by the Danish Data Protection agency (protocol: SJ567).

### Setting

During the perioperative period, patients followed the standard of care for colon cancer in a setting of Enhanced Recovery After Surgery (ERAS), which has been described in detail for the department elsewhere [30]. There were no restrictions on pain management, and all patients were encouraged to take their regular medication after surgery. The choice of anesthetics was determined at a pre-anesthesia interview, and patients received universal anesthesia with either Total Intravenous Anesthesia or volatile inhalational. For induction of anesthesia, propofol 2–3 mg/kg and remifentanil or sufentanil were administered. Hereafter, all patients received a single intravenous dose of 240 mg gentamycin and 1 g metronidazole. Patients assigned to Total Intravenous Anesthesia received a continuous infusion of propofol supplemented remifentanil 0.5 μg/kg/min. Patients assigned to volatile inhalation received sevoflurane to a minimum alveolar concentration of 0.7–1.2 and remifentanil or repeated boli of sufentanil. Prior to extubation, ondansetrone 4 mg, sufentanil 0.4–0.6 μg/kg, and 1 g of paracetamol was given. Ropivacaine, 20 mL, was administered locally in the wounds.

### Data collection and processing

Demographic data was collected through the electronic patient charts including age, gender, smoking status, body mass index (BMI), American Society of Anesthesiologist (ASA) scores, and Charlson Comorbidity Index. The UICC stage was based on pre-operative CT scans and histology results. Blood samples were taken the day prior to surgery, and approximately 24 h after surgery. Samples were collected in serum separation gel-tubes and left undisturbed at room temperature for 30 min to allow clotting. Hereafter, samples were centrifuged at 2330 g at 4^o^ C for 10 min to remove the clot. The resulting supernatant was immediately transferred into Eppendorf tubes and kept at -80 °C until analysis.

### Cell culture

The wild type human colon cancer cell lines Caco-2, DLD-1, SW480, LoVo, LS174T and a CDX2 inducible LS174T cell line were used in this study. The CDX2 inducible LS174T cell line is genetically modified and are CDX2 knockout but contain inducible elements that enable activation of CDX2 expression by addition of doxycycline to the growth media [31]. LS174T cell lines were obtained from Assoc. Prof. Eric Paul Bennett. All cell lines were cultured in Dulbecco’s Modified Eagle’s Medium (DMEM) with Ultraglutamine with 4.5 g/L Glucose (Lonza, Basel, Switzerland) supplemented with 10% Fetal Bovine Serum (HyClone by Fisher Scientific, Waltham, MA, USA) and Penicillin (100 units/mL) Streptomycin (100 μg/mL) (Gibco by Life Technologies, Carlsbad, CA, USA) The cell cultures were incubated at 37 °C in 5% CO2 and passaged every 3–4 days. The LS174T cells with inducible CDX2 were cultured in media with or without 4 ng/ml doxycycline to induce CDX2 expression.

### Adhesion measurement

Real-Time Cell-Analysis (RTCA) iCELLigence (ACEA Biosciences, San Diego, CA, USA) was used to measure cell adhesion. The RTCA iCELLigence instrument uses electrical impedance as a readout to monitor changes in the cellular phenotype. The cell culture plates used in the instrument have electrodes placed at the bottom of each well, and cells attaching to the electrodes will lead to an increase in electrical impedance. The relative change in the electrical impedance is recorded as a dimensionless value termed Cell Index. The RTCA iCELLigence was set up using E-Plate L8 PET (ACEA Biosciences, San Diego, CA, USA) and cells in DMEM containing either 7% pre- or postoperative serum were added to each well in quadruplicates. For the LS174T cell line, 2*10^4^ cells were seeded in each well. For the Caco-2 cell line, 5*10^3^ cells were seeded in each well, while for the DLD-1, SW480, and LoVo, 1*10^4^ cells were seeded in each well. For LS174T cell with inducible CDX2, 2*10^4^ cells with or without 4 ng/ml doxycycline induced CDX2 expression were seeded in replicates in the E-plate L8 PET. The impedance was measured every 5 min and the difference in Cell Index at 60 min between cells seeded in preoperative and postoperative serum was calculated.

### Western blot

Cells for protein extraction were seeded in 6-well plates at 5*10^5^ cells/well. After 24 h media was changed and LS174T cells with inducible CDX2 were added media with or without doxycycline. Cells were lysed after 72 h of doxycycline treatment by rising with cold PBS and incubated 5 min with 150 μl/well 1x RIPA lysis buffer (1x PBS, 300 mM NaCl, 1% Tergitol NP-40, 0.1% SDS, 0.5% 7-Deoxycholic acid sodium salt, 0.5 μM EDTA pH 8.0) with freshly added 1 mM DTT and 2 μl/ml protease inhibitor mix p8340 (Sigma-Aldrich, St. Louis, MO, USA). Lysate was centrifuged for 15 min at 12.000 g and 4 °C. Supernatant was stored at − 20 °C. Protein concentration was determined by Bradford analysis (Bio-Rad, Hercules, CA, USA).

For the analysis, 10 μg protein was mixed 1:4 (v/v) with Bolt loading buffer and 1:10 (v/v) with Bolt sample reducing agent (Thermo Fisher Scientific, Waltham, MA, USA). Samples were incubated at 95 °C for 5 min and loaded on a Bolt 4–12% Bis-Tris Plus gel (Thermo Fisher Scientific, Waltham, MA, USA) PageRuler prestained protein ladder was used as marker (Thermo Fisher Scientific, Waltham, MA, USA). SDS-PAGE was performed in 1X Bolt MOPS running buffer (Thermo Fisher Scientific, Waltham, MA, USA) for 30 min at 25 V, then 60 min at 120 V. The gel was transferred by wet-electrotransfer to PVDF membrane for 60 min at 25 V in 1X NuPage transfer buffer (Thermo Fisher Scientific, Waltham, MA, USA). The membrane was blocked with dry skim milk diluted to 5% in Wash buffer (1X TBS with 0.1% Tween-20) for 1 h at room temperature. The membrane was washed with Wash buffer 5 times for 3 min and incubated overnight at 4 °C with primary antibody diluted in 2.5% skim milk in Wash buffer. The membrane was then washed 5 times for 3 min and incubated with diluted secondary antibody for 1 h at room temperature. Before visualization, the membrane was washed 5 times for 3 min and then visualized by incubating with the ECL solution SuperSignal West Dura Extended Duration Substrate for 5 min (Thermo Fisher Scientific, Waltham, MA, USA). Antibodies used: CDX2 1:1000 (BioGenex, Freemont, CA, USA, MU392A-UC); Vinculin 1:5000 (Abcam, Cambridge, UK, ab129002); Goat anti-rabbit HRP 1:10,000 (Thermo Fisher Scientific, Waltham, MA, USA, 32260); Goat anti-mouse HRP 1:10,000 (Thermo Fisher Scientific, Waltham, MA, USA, 32230).

### Statistical analysis

The paired Wilcoxon signed-rank test was used to determine statistical differences in the adhesion of cells with pre- and postoperative serum and the level of statistical significance was set at *p*-values < 0.01. The RTCA iCELLigence data analysis software 1.0 and Graphpad Prism 8 software were used for statistical analysis.

## Results

In total, 38 patients were enrolled in the study. Seven patients were excluded due to post-operative complications and one patient was excluded due to benign disease. A total of 30 patients, 19 male and 11 female, went through laparoscopic colon cancer surgery within an ERAS setting and were included in the study (see Table 1 for patient demographics). According to UICC staging [32], patients were diagnosed with stage I-III cancer. The patients had an ASA score [33] ranging from I to III and had between 0 and 2 in WHO Performance Status [34]. None of the patients had visible metastasis preoperatively. Serum from blood samples drawn on the day prior to surgery and the day after surgery was used for the analysis of adhesion.

**Table 1:** Demographics for patients undergoing laparoscopic colonic resection for colon cancer

Age, mean (SD)		67,6 (8,8)
**Gender n (%)**	Male	19 (63,3)
	Female	11 (36,7)
**ASA-score n (%)**	1	3 (10,0)
	2	24 (80,0)
	3	3 (10,0)
**BMI n (%)**	< 18,5	1 (3,3)
	18.5–24.9	12 (40,0)
	25–29.9	8 (26,7)
	> 30	9 (30,0)
**Smoking n (%)**	Current smoker	5 (16,7)
	Former smoker	13 (43,3)
	Never smoker	12 (40,0)
**Alcohol (drinks/week) n (%)**	0–14/21	25 (83,3)
	> 14/21	5 (16,7)
**Charlson Comorbidity Index n (%)**	0	18 (60,0)
	1	6 (20,0)
	2	3 (10,0)
	Missing	3 (10,0)
**WHO Performance status n (%)**	0	25 (83,3)
	1	3 (10,0)
	2	2 (6,7)
**UICC n (%)**	1	10 (33,3)
	2	12 (40,0)
	3	8 (26,7)
**Anesthesia n (%)**	Intravenous	20 (66,7)
	Inhalation	10 (33,3)
**Laparoscopic procedure n (%)**	Right hemicolectomy	9 (30,0)
	Transverse colectomy	1 (3,3)
	Left hemicolectomy	1 (3,3)
	Sigmoidectomy	18 (60,0)
	Complete colectomy	1 (3,3)

*ASA* American Society of Anesthesiologist Score, *BMI* Body Mass Index, *UICC* Union for International Cancer Control

**Fig. 1:**
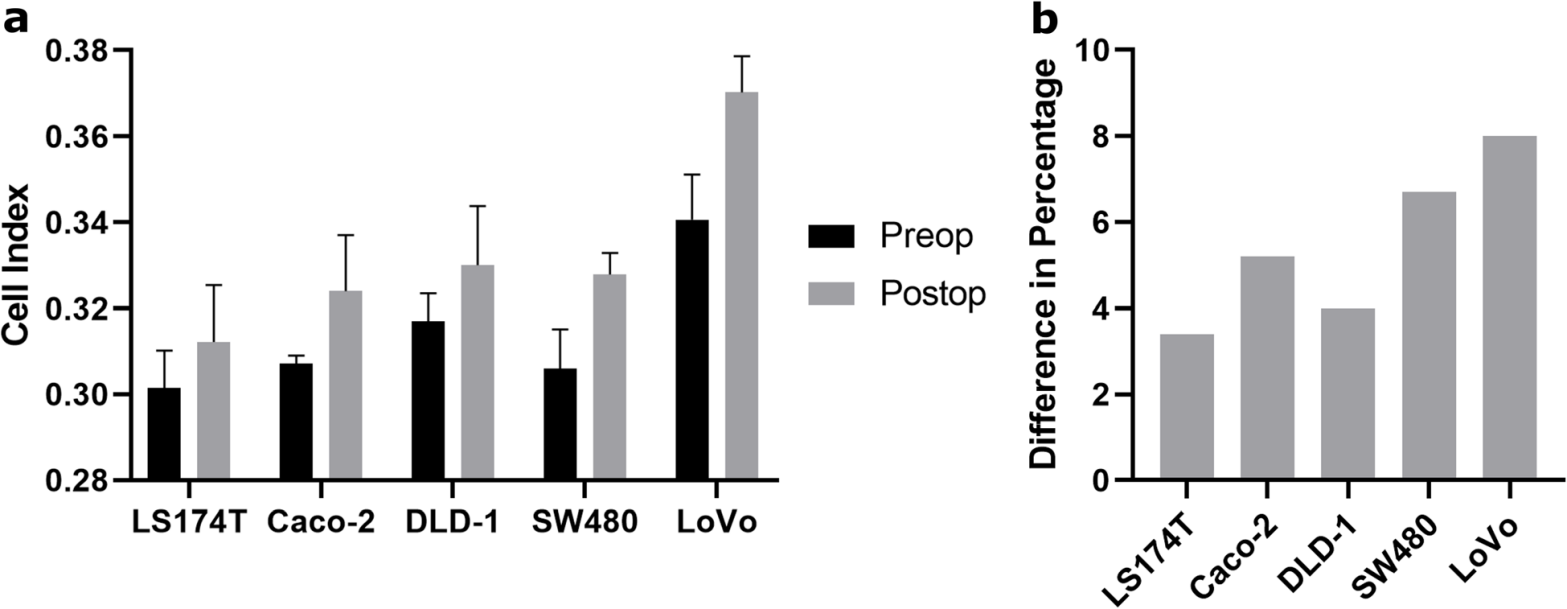
Adhesion measurements of five different colon cancer cell lines in pre- or postoperative patient serum **a.** Cell adhesion of LS174T, Caco-2, DLD-1, SW480, and LoVo cells seeded in media with pre- or postoperative serum from one patient was measured. Mean Cell Index at 60 min is shown, *n* = 4. **b.** The difference in percentage between adhesion ability of cells seeded in postoperative serum compared to preoperative serum at 60 min was calculated for each cell line. The positive bars (grey) indicate higher adhesion in cells in postoperative serum compared to cells in preoperative serum

Culturing five different colon cancer cell lines, LS174T, Caco-2, DLD-1, SW480, and LoVo, in media supplemented with perioperative serum from a single patient, showed increased adhesion abilities in cells seeded in postoperative serum compared to preoperative serum for all cell lines (Fig. 1a). The difference in Cell Index in percentage at 60 min varied from 3.5% in the LS174T cell line to 8.0% in the LoVo cell line (Fig. 1b). While all the cell lines showed varied extent of increase in adhesion in postoperative serum, we chose the LS174T cell line for testing our entire patient cohort consisting of 30 patients. This cell line was chosen as a genetically modified clone has been produced, which contains inducible elements that control the expression of CDX2 [31]. As a result, the cells do not express CDX2 without being induced. To our knowledge, this is the only colon cancer cell line still viable with complete depletion of CDX2 expression. In other CDX2 positive colon cancer cell lines, CDX2 acts as a linage survival gene that cannot be inactivated [35].

**Fig. 2:**
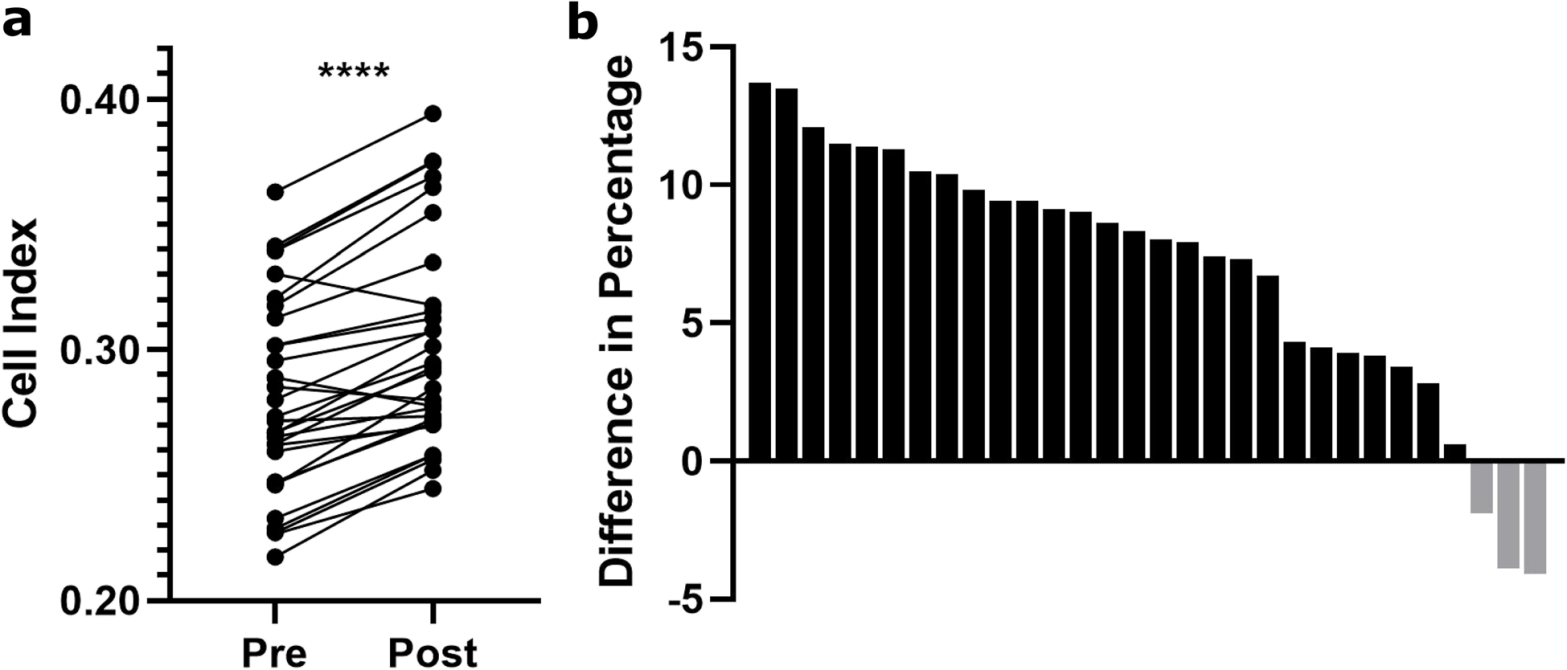
Adhesion measurements in wild type LS174T cells **a**. The Cell Index for wild type LS174T cells seeded in pre- and postoperative serum was measured for each patient. Mean results at 60 min for pre- and postoperative cell adhesion for each patient is shown. ****p < 0.0001. **b.** The difference in percentage in adhesion at 60 min was calculated for each patient**.** The positive bars (black) indicate patients with higher adhesion in cells in postoperative compared to preoperative serum, while the negative bars (grey) indicate patients with higher adhesion in cells in preoperative compared to postoperative serum

When investigating our cohort of 30 patients a significant difference in cell adhesion, with increased adhesion in wild type LS174T cells seeded in postoperative serum compared to preoperative serum was observed. A difference between the pre- and postoperative samples could be observed 20 min after seeding, and at 60 min the cells had adhered to the surface and no further increase in adhesion could be observed. The Cell Indexes at 60 min were for 26 out of 30 patients higher in the postoperative sample compared to the preoperative sample (*p* < 0.0001) (Fig. 2a). Cell Indexes were slightly lower for three patients in the postoperative serum (Fig. 2b). The sera from one patient gave the same Cell Index before and after surgery.

**Fig. 3:**
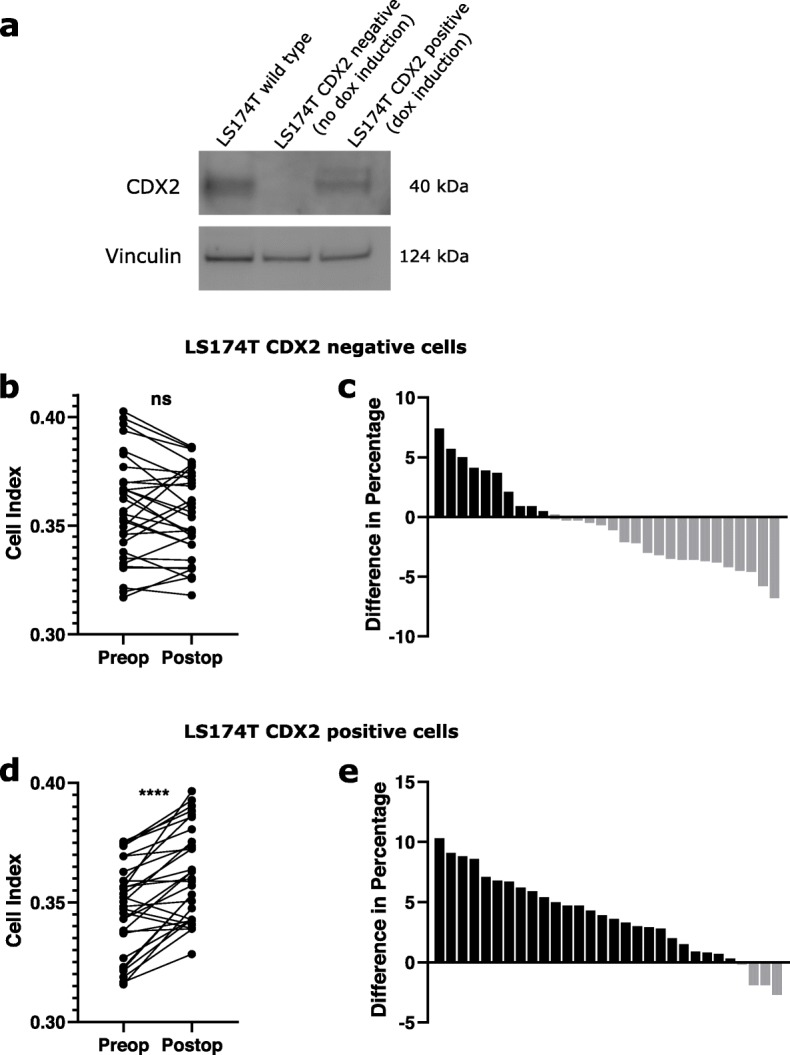
Adhesion measurements in CDX2 inducible LS174T cells **a.** CDX2 protein expression was compared using western blotting. Cell lysate from LS174T wild type cells and LS174T cells with inducible CDX2 with or without doxycycline treatment was used in the analysis. Vinculin was used as a control. Bands are from the same gel **b.** The Cell Index for CDX2 negative LS174T cells seeded in pre- and postoperative serum was measured. Mean results at 60 min for pre- and postoperative cell adhesion for each patient is shown. n.s. = not significant. **c.** The difference in percentage in adhesion at 60 min was calculated for each patient**.** The positive bars (black) indicate patients with higher adhesion in postoperative compared to preoperative serum, while the negative bars (grey) indicate patients with higher adhesion in preoperative compared to postoperative serum. **d.** The Cell Index for CDX2 positive LS174T cells seeded in pre- and postoperative serum was measured. Mean results at 60 min for pre- and postoperative cell adhesion for each patient is shown. ****p < 0.0001. **e.** The difference in percentage in adhesion at 60 min was calculated for each patient**.** The positive bars (black) indicate patients with higher adhesion in postoperative compared to preoperative serum, while the negative bars (grey) indicate patients with higher adhesion in preoperative compared to postoperative serum

To investigate the role of CDX2 in cell adhesion, the colon cancer cell line LS174T with inducible CDX2 was used. This cell line has previously been used to study the effect of CDX2 on intestinal transcriptional regulation [36–38]. Western blotting analysis of the LS174T wild type and LS174T with inducible CDX2 cells was performed to detect CDX2 levels. Results show no CDX2 expression in the LS174T with inducible CDX2 when not treated with doxycycline (Fig. 3a). When treated with doxycycline, expression of CDX2 was re-established. Vinculin was used as a control to measure total protein loaded. When seeding CDX2 negative LS174T cells in pre- and postoperative patient serum no difference in adhesion between the two groups was seen (*p* = 0.21) (Fig. 3b). Out of the 30 patient samples, only 11 had increased adhesion for cells seeded in postoperative serum compared to preoperative serum (Fig. 3c). However, when the cells had induced CDX2 at wild type levels, the results resembled those seen in the wild type LS174T cells, with significantly increased adhesion in postoperative serum compared to preoperative serum (*p* < 0.0001) (Fig. 3d). Twenty-six patients out of 30 showed increased adhesion for cells seeded in postoperative serum compared to preoperative serum (Fig. 3e).

**Fig. 4:**
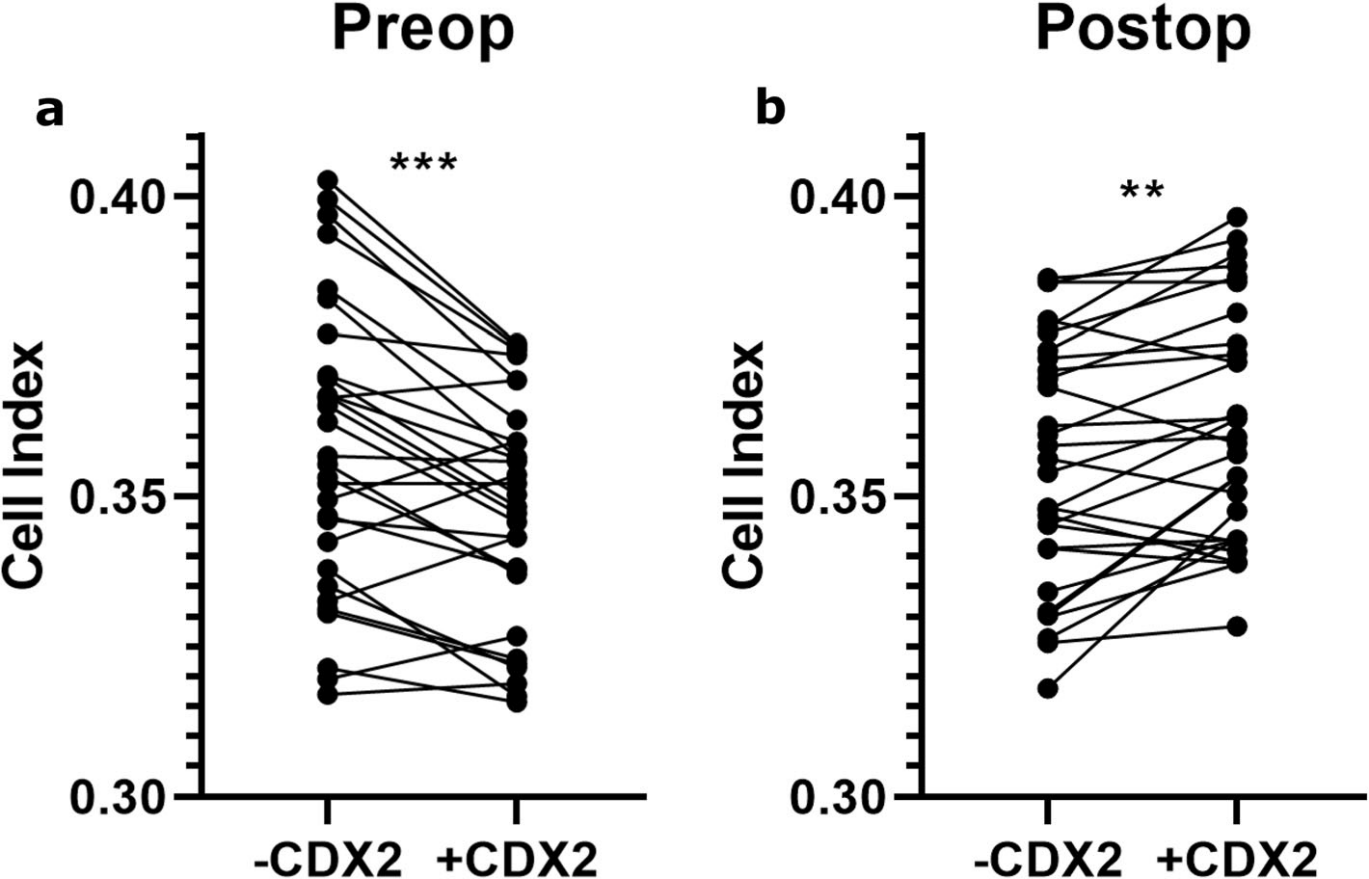
Adhesion measurement of CDX2 inducible LS174T cells in pre- and postoperative serum **a.** The Cell Index for CDX2 negative and CDX2 positive inducible LS174T cells seeded in preoperative serum was measured. Mean results at 60 min for each patient is shown. ***p < 0.001. **b.** The Cell Index for CDX2 negative and CDX2 positive inducible LS174T cells seeded in postoperative serum was measured. Mean results at 60 min for each patient is shown. **p < 0.01

When comparing cell adhesion in cells treated with preoperative serum samples, there was a significant increase in adhesion in the CDX2 negative cells compared to the CDX2 positive cells (*p* < 0.001) (Fig. 4a). For the cells treated with postoperative serum, the CDX2 positive cells had significantly increased adhesion compared to the CDX2 negative cells (*p* < 0.001) (Fig. 4b).

## Discussion

In this study, we established an in vitro method for measuring the effect of perioperative factors on the adhesion ability of the LS174T colon cancer cell line using serum from patients undergoing colon cancer surgery. Commonly used methods for cell adhesion assays typically include staining attached cells and using fluorescence for endpoint measurements [39–41], but by using the method developed in this paper, it is possible to monitor real-time cell adhesion for the entire adhesion period. While this method does not allow us to distinguish between initial sedimentation, cell attachment, cell spreading and stable cell adhesion, the mentioned are all part of the passive cell adhesion process [42]. Investigating the adhesion abilities of the cells on a surface that more resembles the in vivo biological surface cancer cells interact with during metastasis may provide further insight to the adhesion process examined in this study.

Our study identified significantly increased cell adhesion abilities in five different colon cancer cell lines in postoperative serum, and further investigation using genetically modified LS174T cells showed this increase in adhesion to be eliminated by lack of CDX2 expression. This indicates that the absence of CDX2 expression results in reduced cancer cell adherence, and that fluctuation of CDX2 levels in cancer cells could be important in the metastatic process of colon cancer cells.

CDX2 has been shown to regulate the expression of a number of claudins [43, 44], a critical component of the tight junctions in epithelial cells. Aberrant expression of claudins has been seen in a variety of cancers, and it has been hypothesized that reduced claudin expression promotes tumorigenesis and metastasis by increasing the motility and invasion of cancer cells [9]. Studies have shown that reduced expression of claudin-1 is a predictor of poor prognosis and reduced disease-free survival [45–47], and that knockdown of claudin-1 expression in colon cancer cell lines significantly increase cell invasiveness [45]. Reduced expression of claudin-7 has been shown to be an early event in colorectal carcinogenesis [48], and downregulation of claudin-7 promotes EMT [49, 50]. Expression of claudin-23 has been shown to be downregulated in tumour tissue and downregulation is associated with shorter overall survival in patients with colorectal tumours [51]. Furthermore, CDX2 has been shown to mediate E-selectin ligand expression in colon cancer cells [52], a crucial component in the attachment of cancer cells to distant tissues during metastasis [10].

Exogenous CDX2 expression has been shown to be associated with reduced cell invasion in Lovo cells transfected with CDX2 overexpression plasmid [53], indicating that CDX2 may play a role in other metastatic processes besides adhesion. Other components have also been shown to influence the metastatic processes of adhesion, invasion and migration, such as the G-protein coupled receptor 55 [54], and the C-type lectin DC-SIGNR [55].

The observed difference in adhesion property between cells in pre- versus postoperative serum is most likely due to factors released into the bloodstream in patients during or after surgery. Previous studies have shown that pro-inflammatory cytokines mediate the adhesion of cancer cells to mesothelial and endothelial monolayers in vitro [13, 14]. Changes in expression of cell adhesion molecules in colon cancer cells have been associated with progression of cancer. This alteration in adhesion molecules could potentially facilitate the adhesion enabling intravasation as well as extravasation and may be part of organ selectivity in metastatic processes [15]. Furthermore, changes in expression of adhesion molecules could also affect postoperative cancer cell survival, as circulating tumour cells are vulnerable and depend on fast attachment in order to survive [2].

The underlying mechanisms of the interaction between the cellular adhesion molecules and factors in the patient serum has not yet been determined. However, when seeding the cells in patient serum, we can already measure altered adhesion abilities between cells in pre- vs postoperative serum 20 min after seeding. This rapid response indicates that factors in the patient serum directly affects the adhesion molecules already expressed on the surface of the cells or in the cytoplasm. Previously, RNA sequencing of the CDX2 inducible LS174T cell line used in our study showed altered RNA levels of several integrins, including integrin α3, α6, β4,and β6, in cells without CDX2 expression compared to wild-type LS174T cells [31]. Given the importance of the postoperative elevated adhesion and its possible correlation with recurrence, an identification of the precise mechanisms behind the interaction may provide valuable knowledge in reducing disease recurrence.

## Conclusions

CDX2 expression is low in invasive colorectal cancer cells but is restored in metastases to a level corresponding to that of the primary tumour [26]. Our results show that CDX2 expression influences the adhesion ability of cultured colon cancer cells, and indicates that adjustments in CDX2 expression levels in cancer cells during EMT and MET is vital in the metastatic process of colon cancer. In conclusion, we demonstrate an in vitro method for measuring the effect of perioperative factors on the adhesion ability of the LS174T colon cancer cell line using serum form patients undergoing colon cancer surgery, and we demonstrate a differential effect on adhesion depending on CDX2 expression. If results from the method developed in this study can be shown to correlate with clinical oncological outcomes, the method may be applied in studies examining perioperative interventions in respect to their effect on short and long-term oncological outcomes after surgery.

## Data Availability

The datasets used and/or analysed during the current study are available from the corresponding author on reasonable request.

## Abbreviations

ASA: American society of Anaesthesiologists
BMI: Body Mass Index
CDX2: Caudal Type Homeobox 2
DMEM: Dulbecco’s Modified Eagle’s Medium
EMT: Epithelial-to-Mesenchyme Transition
ERAS: Enhanced Recovery After Surgery
MET: Mesenchyme-to-Epithelial Transition
RCTA: Real-Time Cell-Analysis
SSR: Surgical Stress Response
UICC: Union for International Cancer Control
